# Involvement of the *hemP-hemA-smlt0796-smlt0797* Operon in Hemin Acquisition by Stenotrophomonas maltophilia

**DOI:** 10.1128/spectrum.00321-22

**Published:** 2022-06-06

**Authors:** Yung-Luen Shih, Cheng-Mu Wu, Hsu-Feng Lu, Li-Hua Li, Yi-Tsung Lin, Tsuey-Ching Yang

**Affiliations:** a Department of Pathology and Laboratory Medicine, Shin Kong Wu Ho-Su Memorial Hospital, Taipei, Taiwan; b School of Medical Laboratory Science and Biotechnology, Taipei Medical University, Taipei, Taiwan; c School of Medicine, College of Medicine, Fu-Jen Catholic University, New Taipei City, Taiwan; d Department of Biotechnology and Laboratory Science in Medicine, National Yang Ming Chiao Tung University, Taipei, Taiwan; e Department of Medical Laboratory Science and Biotechnology, Asia University, Taichung, Taiwan; f Department of Pathology and Laboratory Medicine, Taipei Veterans General Hospital, Taipei, Taiwan; g School of Medical Laboratory Science and Biotechnology, College of Medical Science and Technology, Taipei Medical University, Taipei, Taiwan; h Division of Infectious Diseases, Department of Medicine, Taipei Veterans General Hospital, Taipei, Taiwan; i School of Medicine, National Yang Ming Chiao Tung University, Taipei, Taiwan; University of Manitoba

**Keywords:** HemA, HemP, hemin, TonB-dependent receptor

## Abstract

The hemin acquisition system of Stenotrophomonas maltophilia was elucidated in this study. To identify the TonB-dependent outer membrane receptor for hemin in S. maltophilia, the hemin acquisition systems of Pseudomonas aeruginosa were referenced. PhuR, HasA, and HxuA are three known TonB-dependent outer membrane receptors involved in hemin acquisition by P. aeruginosa. Thus, HemA (Smlt0795) and Smlt2937, the orthologs of PhuR and HasA/HxuA in S. maltophilia, were first considered. KJΔEnt, a stenobactin-null strain, was used as the parental strain for the hemin utilization assay. Deletion of *hemA*, but not Smlt2937, of KJΔEnt impaired hemin acquisition under iron-depleted conditions, indicating that HemA is the TonB-dependent receptor for hemin uptake. The *hemA* gene is a member of the *hemP-hemA-smlt0796-smlt0797* operon, whose expression was upregulated in a *fur* mutant and under iron-depleted conditions. The contribution of the *hemP-hemA-smlt0796-smlt0797* operon to hemin acquisition was investigated by in-frame deletion mutant construction and hemin utilization assays. Inactivation of *hemP*, *smlt0796*, and *smlt0797* of KJΔEnt insignificantly affected hemin acquisition under iron-depleted conditions. However, *hemP* deletion in a *fur* mutant increased hemin acquisition under iron-depleted conditions. Collectively, we revealed that (i) HemA likely functions as the outer membrane receptor for hemin uptake; (ii) HemP, a predicted transcriptional factor, apparently functions as a repressor of the expression of the *hemA* transcript; and (iii) in a *fur* mutant, HemP has a negative impact on hemin acquisition under iron-depleted conditions.

**IMPORTANCE** Stenotrophomonas maltophilia is an emerging multidrug-resistant opportunistic pathogen, increasing the difficulty of treatment of this infection. Iron is a critical element for bacterial viability. Heme is the most abundant iron source in the human host; thus, heme is the major iron source for a pathogen in the infection niche. Blocking iron acquisition from heme can be an alternative strategy to control S. maltophilia infection. Although several hemin acquisition systems have been reported in various pathogens, very little is known about the hemin acquisition systems of S. maltophilia. By in-frame deletion mutant construction and hemin utilization assays, we demonstrated that HemA (Smlt0795) is the TonB-dependent outer membrane receptor for hemin uptake and that HemP (Smlt0794), a predicted transcriptional factor, had a negative impact on hemin acquisition in a *fur* mutant. The negative regulatory role of HemP in hemin acquisition is first reported.

## INTRODUCTION

Iron is an essential nutrient for microorganisms. During infection, a coordinated human host cell response limits the availability of iron to the microbes, a process referred to as nutritional immunity (1). To escape the stress of nutritional immunity that is imposed by the host, pathogens have evolved several strategies to obtain iron during infection. Heme accounts for the majority of the iron pool in vertebrates and is the largest source of iron for bacterial pathogens within the host (2). Free heme is not readily available in the host, as the majority is located in hemoglobin and sequestered within erythrocytes (3). To facilitate the use of heme, certain bacteria secrete hemolysin or hemoglobin protease to degrade hemoglobin, which releases hemin (4). Gram-negative bacteria are generally equipped with TonB-dependent outer membrane proteins (OMPs) as specific receptors for the direct uptake of hemin from host cells. In addition, certain bacteria can synthesize and secrete hemophores, which are small proteins with a high affinity for heme, and take up the hemophore-heme complex via specific TonB-dependent receptors (5). TonB-dependent receptors, a family of β-barrel proteins, are involved in the uptake and transport of ferric iron-associated complexes. The transport process requires energy and a complex of three inner membrane proteins, TonB-ExbB-ExbD, to transduce this energy to the outer membrane. Periplasmic hemin-binding proteins shuttle hemin from the periplasm into the cytoplasm via inner membrane ABC-type transporters such as HmuU of Ensifer meliloti (6). In the cytoplasm, hemin is bound to cytoplasmic hemin-binding proteins and is subsequently degraded by heme oxygenase (7).

There are three known ways for Pseudomonas aeruginosa to utilize hemin as an iron source for growth under iron-depleted conditions: the Phu (Pseudomonas heme uptake) system, the Has (heme assimilation) system, and the Hxu system (8, 9). The Phu system is the major mechanism by which P. aeruginosa can directly take up hemin from the external environment via the PhuR receptor when cytoplasmic iron is depleted (8). The Has system is a hemophore-dependent mechanism. When P. aeruginosa grows under iron-depleted conditions, hemophores are endogenously synthesized and are secreted into the extracellular environment. Hemophores specifically capture hemins, and the hemophore-hemin complex is taken up via the HasR receptor (8). The Hxu system was recently identified and is capable of the direct uptake of hemin as an iron source. However, in P. aeruginosa, it has been verified that the Hxu system plays a major role in signaling the presence of hemin in the extracellular environment and a minor role in hemin acquisition (9).

The ferric uptake regulator (Fur) is a transcriptional regulator that participates in the regulation of iron homeostasis in several pathogens. Under iron-replete conditions, Fur utilizes Fe^2+^ as a corepressor, and the Fur-Fe^2+^ complex binds to a specific sequence, the Fur box, consequently repressing the transcription of iron-responsive genes and operons (10). When the intracellular ferrous iron level is too low to bind with Fur, free-form Fur dissociates from the Fur box, allowing the RNA polymerase to bind and initiate the transcription of genes related to the acquisition and utilization of iron sources (11).

Stenotrophomonas maltophilia is a Gram-negative, free-living bacterium commonly found in soil, water, and plants. This bacterium is increasingly recognized as an opportunistic pathogen in immunocompromised and cystic fibrosis patients (12). S. maltophilia infections are difficult to treat due to the intrinsic and acquired resistance of the bacterium to several antibiotics such as aminoglycosides, β-lactams, and macrolides (13). The bacterium can colonize many different host environments, which may be driven by its ability to scavenge iron during infection. The putative iron acquisition systems of S. maltophilia have been pointed out by Kalidasan et al. using an *in silico* approach, mainly siderophore- and/or heme-mediated iron acquisition systems (14). The siderophore system of S. maltophilia has been well characterized. In response to iron-depleted stress, stenobactin is synthesized in the cytoplasm by enzymes encoded by the *entCEBB′FA* gene cluster and exported into the extracellular environment for ferric iron acquisition (15, 16). However, the understanding of the strategies used by S. maltophilia to acquire heme-containing iron is still limited. In this study, we present the first characterization of *hemP* (Smlt0794) and *hemA* (Smlt0795) in hemin acquisition by S. maltophilia. HemA is the TonB-dependent outer membrane receptor of hemin. We also show a unique property of HemP as a transcriptional factor in repressing *hemP-hemA-smlt0796-smlt0797* operon expression, which is distinct from the functional homologs, HemP/HmuP, in other heme-utilizing bacteria (17–20).

## RESULTS

### S. maltophilia can utilize hemin as an iron source to support growth under iron-depleted conditions.

**FIG 1:**
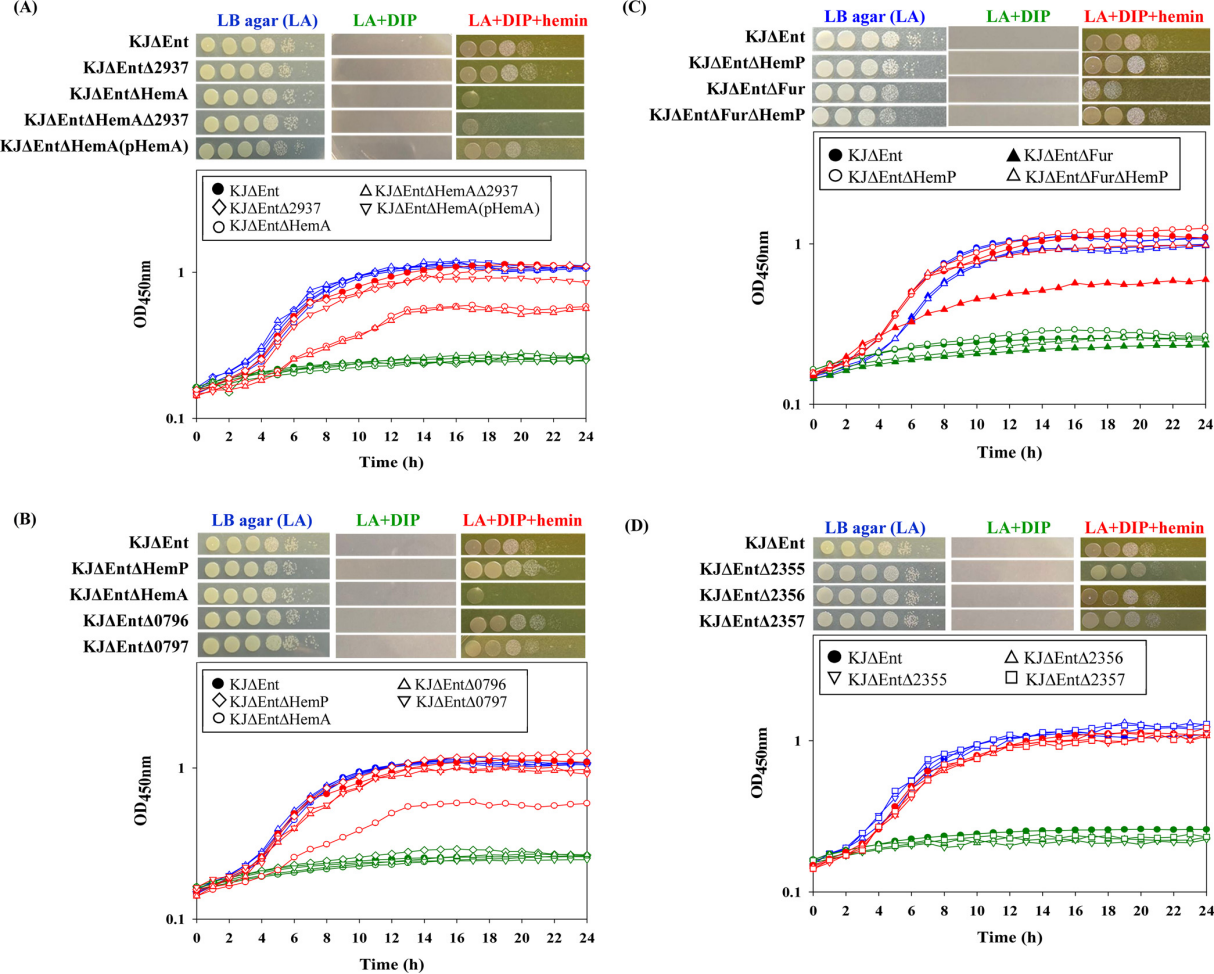
Cell viability and bacterial growth under iron-replete conditions, iron-depleted conditions, and iron-depleted conditions with hemin as the sole iron source. For cell viability, logarithmic-phase bacterial cells tested at 2 × 10^5^ CFU/μL were 10-fold serially diluted. Five microliters of the bacterial suspension was spotted onto LB agar plates as indicated. The growth of bacterial cells was recorded after a 24-h incubation at 37°C. The concentrations of DIP and hemin added are 50 μg/mL and 150 μM, respectively. For bacterial growth, bacterial cells cultured overnight were inoculated into the indicated medium at an initial OD_450_ of 0.15. Bacterial growth was recorded by monitoring the OD_450_ for 24 h at intervals of 1 h. Blue, green, and red lines indicate iron-replete conditions (LB broth), iron-depleted conditions (LB plus DIP), and iron-depleted conditions with hemin as the sole iron source (LB plus DIP and hemin), respectively. All experiments were performed at least three times, and one was selected as a representative experiment. (A) Roles of *smlt2937* and *hemA* (*smlt0795*) in hemin acquisition. (B) Role of the *hemP-hemA-smlt0796-smlt0797* operon in hemin acquisition. (C) Roles of *fur* and *hemP* in hemin acquisition. (D) Roles of *smlt2355*, *smlt2356*, and *smlt2357* in hemin acquisition.

We used iron utilization assays to assess hemin utilization of S. maltophilia KJ under iron-depleted conditions. To avoid the bias caused by stenobactin, KJΔEnt, a stenobactin-null mutant carrying *entC* and *entF* deletions (21), was used. KJΔEnt was unable to grow in 50 μg/mL 2,2′-dipyridyl (DIP)-containing Luria-Bertani (LB) agar but grew when 150 μM hemin was included in the medium (Fig. 1A), indicating that S. maltophilia KJ is capable of utilizing hemin as an iron source for growth under iron-depleted conditions.

### Smlt0795 (HemA), rather than Smlt2937, is the TonB-dependent OMP for hemin uptake.

A homolog search of hemophores in the S. maltophilia K279a genome was conducted using the HasA proteins of P. aeruginosa (GenBank accession no. AAG06795), Serratia marcescens (accession no. CAA57068), and Acinetobacter baumannii (accession no. SKV34882) as the queries, but no HasA homolog was identified. Thus, S. maltophilia seems not to synthesize a hemophore for hemin uptake. PhuR (PA4710), HasR (PA3408), and HxuA (PA1302) are three well-known TonB-dependent OMP receptors involved in heme-associated uptake in P. aeruginosa (9). To identify OMPs for hemin uptake in S. maltophilia, we used the PhuR, HasR, and HxuA proteins of P. aeruginosa as queries for a candidate search. The search revealed that the most similar homolog of PhuR in the S. maltophilia genome was Smlt0795 (37% identity and 52% similarity). Smlt0795 was designated *hemA* here based on the following results. In addition, when HasR and HxuA were used as queries, the same candidate, Smlt2937, was revealed. Smlt2937 shared 26% identity and 40% similarity with HasR and 27% identity and 41% similarity with HxuA.

The known roles of PhuR, HasR, and HxuA in hemin uptake (8, 9), together with sequence comparisons, suggested the involvement of *hemA* and Smlt2937 in hemin uptake. To test this hypothesis, we constructed *hemA* and Smlt2937 single- and double-deletion mutants from the stenobactin-null strain KJΔEnt (21), yielding KJΔEntΔHemA, KJΔEntΔ2937, and KJΔEntΔHemAΔ2937. The viability of each mutant and the parental strain (KJΔEnt) was examined in iron-replete medium and iron-depleted medium with and without hemin. All strains tested had comparable growth in LB agar, but growth was stunted in LB agar containing 50 μg/mL DIP. The growth of KJΔEnt was restored when 150 μM hemin was added to DIP-containing LB agar. KJΔEntΔHemA displayed compromised viability in hemin-supplemented medium compared to that of KJΔEnt, but KJΔEntΔ2937 did not (Fig. 1A). Complementation of KJΔEntΔHemA with plasmid pHemA restored growth (Fig. 1A). Furthermore, the deletion of Smlt2397 from the chromosome of KJΔEntΔΗemA did not further compromise viability in hemin-supplemented medium (Fig. 1A), supporting the insignificance of Smlt2397 for hemin uptake. To further quantitatively confirm the role of *hemA* in hemin acquisition, a growth assay in liquid medium was carried out. The involvement of *hemA* in hemin acquisition was also supported (Fig. 1A). Consequently, HemA, rather than Smlt2937, is the major TonB-dependent OMP for hemin uptake in S. maltophilia KJ, which is consistent with the previous prediction proposed by Kalidasan et al. (14).

### Role of the *hemP-hemA-smlt0796-smlt0797* operon in hemin acquisition.

**FIG 2:**
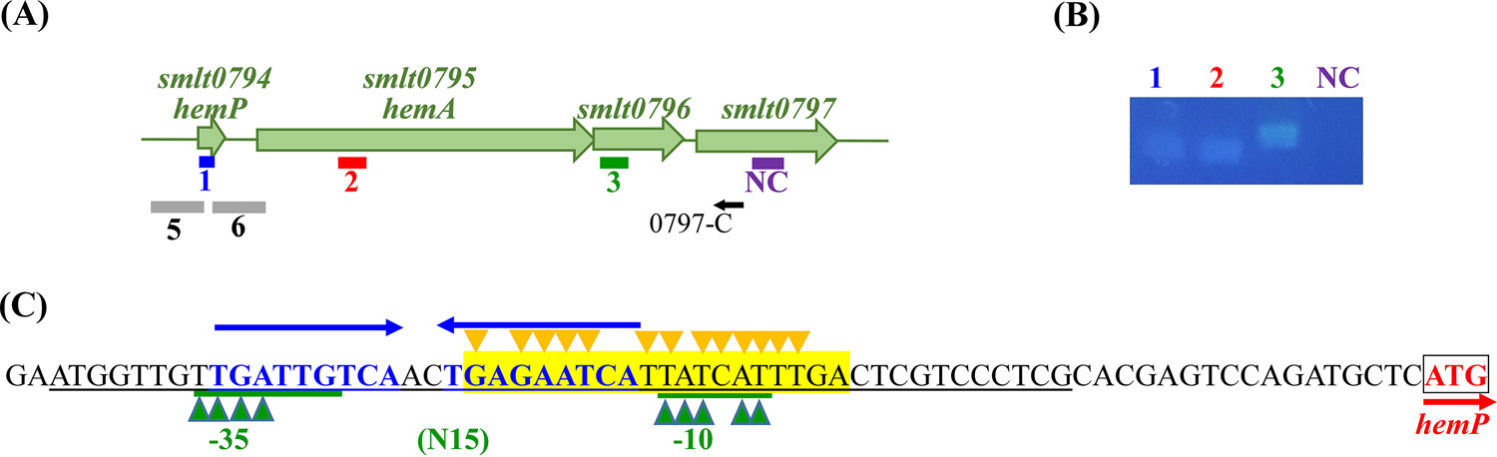
*hemP-hemA-smlt0796-smlt0797* operon verification and expression. (A) Genetic organization of the *hemP-hemA-smlt0796-smlt0797* cluster of S. maltophilia KJ. The orientation of the gene is indicated by an arrow. The black small arrows indicate the locations of the 0797-C primer for reverse transcription. The bars indicate the PCR amplicons obtained using primer sets HemPQ95-F/R (blue), HemAQ93-F/R (red), 0796Q120-F/R (green), and 0797Q108-F/R (purple) and are labeled 1, 2, 3, and NC, respectively. The gray bars indicate the DNA segments cloned into pHemP_xylE_ (labeled 5) and pHemA_xylE_ (labeled 6). (B) Agarose gel electrophoresis of the PCR products. DNA-free RNA collected from KJΔFur underwent reverse transcription using the primer 0797-C. Next, the 0797-C-derived cDNA was used as the template for PCR with the primers indicated. 1, primers HemPQ95-F and HemPQ95-R; 2, primers HemAQ93-F and HemAQ93-R; 3, primers 0796Q120-F and 0796Q120-R; NC (negative control), primers 0797Q108-F and 0797Q108-R. (C) Analysis of the promoter-containing region of the *hemP-hemA-smlt0796-smlt0797* operon. The putative Fur box is marked in yellow, based on the previously reported Fur box sequence (23). The nucleotides that matched the Fur box consensus sequence are indicated with yellow triangles. The predicted −10 and −35 promoter regions and the spacing of the *hemP-hemA-smlt0796-smlt0797* operon are underlined in green. The nucleotides matching the promoter consensus sequence are indicated with green triangles. The putative HemP-binding region is marked in blue, and the inverted repeat sequence is indicated with blue arrows.

**FIG 3:**
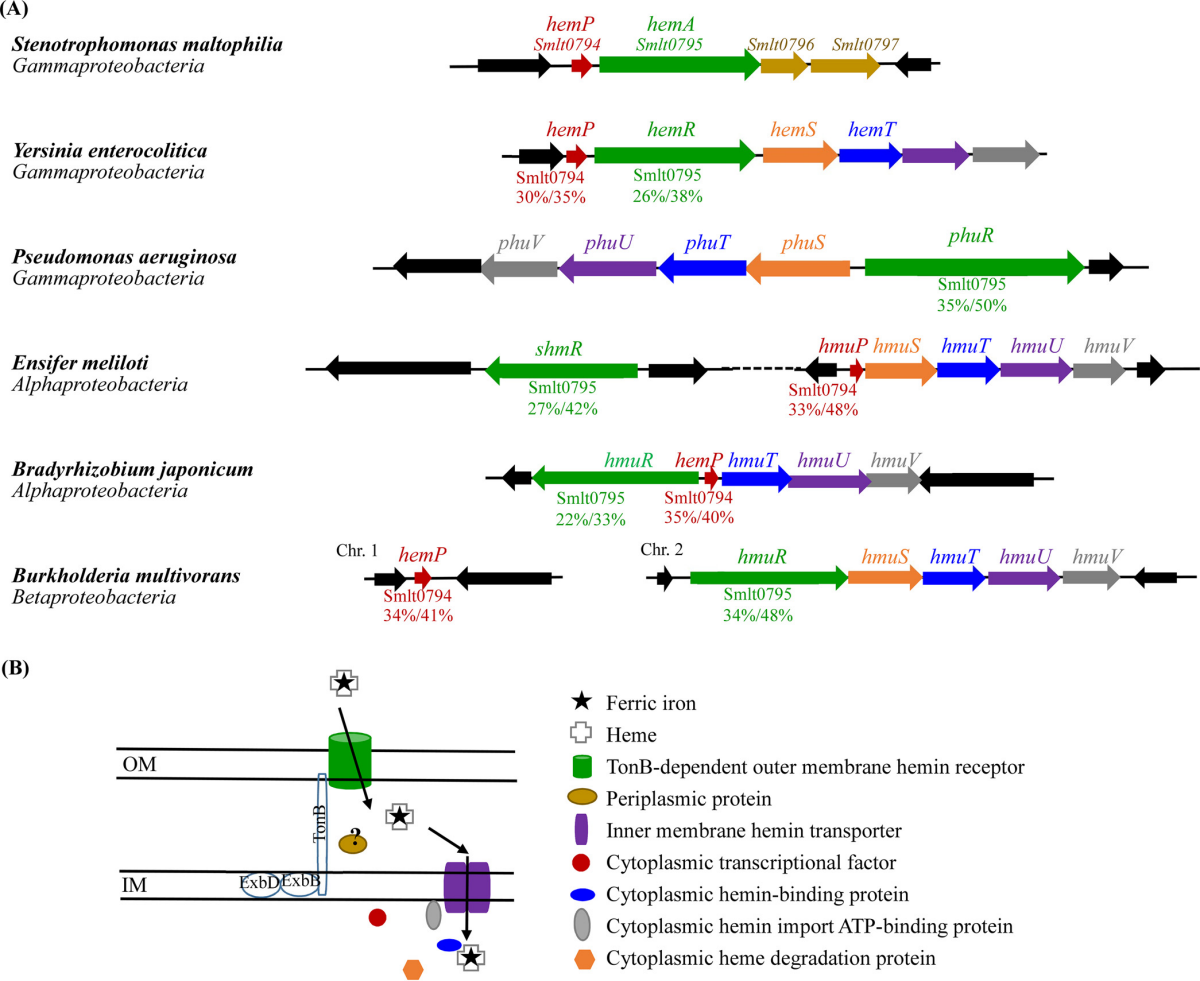
Genetic organizations and cartoon illustration of hemin acquisition systems in S. maltophilia, Y. enterocolitica, P. aeruginosa, *E. meliloti*, B. japonicum, and *B. multivorans*. Genes and proteins are color-coded. Colors represent the putative location and function of the encoded proteins. Red, cytoplasmic transcriptional factor (HemP/HmuP); green, TonB-dependent outer membrane hemin receptor; brown, periplasmic protein; purple, inner membrane hemin permease; blue, cytoplasmic hemin-binding protein; gray, cytoplasmic hemin import ATP-binding protein; orange, cytoplasmic heme degradation protein. (A) Genetic organizations of hemin acquisition systems. (B) Cartoon illustration of a hemin acquisition system. OM, outer membrane; IM, inner membrane.

The TonB-dependent OMP genes are usually located near other genes related to iron uptake. The genomic organization surrounding *hemA* was assessed in the S. maltophilia K279a genome. A four-gene cluster, Smlt0794 to Smlt0797 (Fig. 2A), interested us. The Smlt0794 gene encodes a 66-amino-acid (aa) cytoplasmic protein that shows protein identities of 35%, 33%, 35%, and 34% with HemP of Yersinia enterocolitica, HmuP of Ensifer meliloti, HmuP of Bradyrhizobium japonicum, and HemP of Burkholderia multivorans, respectively (Fig. 3). The HemP and HmuP proteins are transcriptional activators of the genes encoding outer membrane hemin receptors in these heme uptake systems (17–20). Thus, we designated the Smlt0794 gene *hemP* here. HemA is a TonB-dependent outer membrane protein responsible for the uptake of hemin based on our above-mentioned results (Fig. 1A). Smlt0796 and Smlt0797 are predicted to be periplasmic proteins by the CELLO v.2.5 subcellular localization predictor (http://cello.life.nctu.edu.tw/). We also performed signal peptide prediction for Smlt0796 and Smlt0797 with SignalP 6.0 (https://services.healthtech.dtu.dk/service.php?SignalP-6.0). Smlt0797 had a 20-aa predicted signal peptide, but no predicted signal peptide was revealed for Smlt0796.

Reverse transcriptase PCR (RT-PCR) was performed to verify whether the four genes formed an operon. Given that the iron uptake system is generally inactive under iron-replete conditions, we prepared DNA-free RNA from the logarithmic growth phase of KJΔFur, a *fur* isogenic in-frame deletion mutant (16), grown in LB broth. The results indicated that *smlt0794*, *hemA*, *smlt0796*, and *smlt0797* form an operon (Fig. 2B).

To study the function of the *hemP-hemA-smlt0796-smlt0797* operon in hemin utilization, the isogenic in-frame single-deletion mutants were individually constructed in KJΔEnt, yielding KJΔEntΔHemP, KJΔEntΔHemA, KJΔEntΔ0796, and KJΔEntΔ0797. KJΔEntΔHemP, KJΔEntΔ0796, and KJΔEntΔ0797 exhibited growth comparable to that of the parental strain in iron-depleted medium supplemented with hemin (Fig. 1B). The results of growth in liquid medium were consistent with this conclusion (Fig. 1B). Thus, only *hemA*, but not *hemP*, *smlt0796*, and *smlt0797*, is required for hemin uptake in S. maltophilia.

### Comparison of hemin acquisition systems of microorganisms.

Given that HemP homologs have been reported in Y. enterocolitica, E. meliloti, B. japonicum, and B. multivorans and that P. aeruginosa and S. maltophilia are often coisolated from cystic fibrosis patients, the hemin acquisition systems in the five microorganisms were compared to the *hemP-hemA-smlt0796-smlt0797* operon of S. maltophilia.

The conservation of *hemP* homologs in gammaproteobacteria is not widespread, although *hemP* or *hmuP* homologs are highly conserved in alpha- and betaproteobacteria (14) (Fig. 3A). Among gammaproteobacteria, no *hemP* homolog was identified in the genome of P. aeruginosa PAO1, while Y. enterocolitica and S. maltophilia contained *hemP* homologs (Fig. 3A). Usually, hemin utilization-associated genes are adjacently located, such as *hemP-hemRSTUV* in Y. enterocolitica, *phuR-phuSTUV* in P. aeruginosa, *hmuP-hmuSTUV* in *E. meliloti*, *hmuR-hemPTUV* in B. japonicum, and *hmuR-hmuSTUV* in *B. multivorans*. However, no *hmuSTUV* or *phuSTUV* homologs were found in the *hemPA* cluster of S. maltophilia (Fig. 3A). Furthermore, Smlt0796 and Smlt0797 did not display significant identities to the known components of other hemin utilization systems (Fig. 3A).

**FIG 4:**
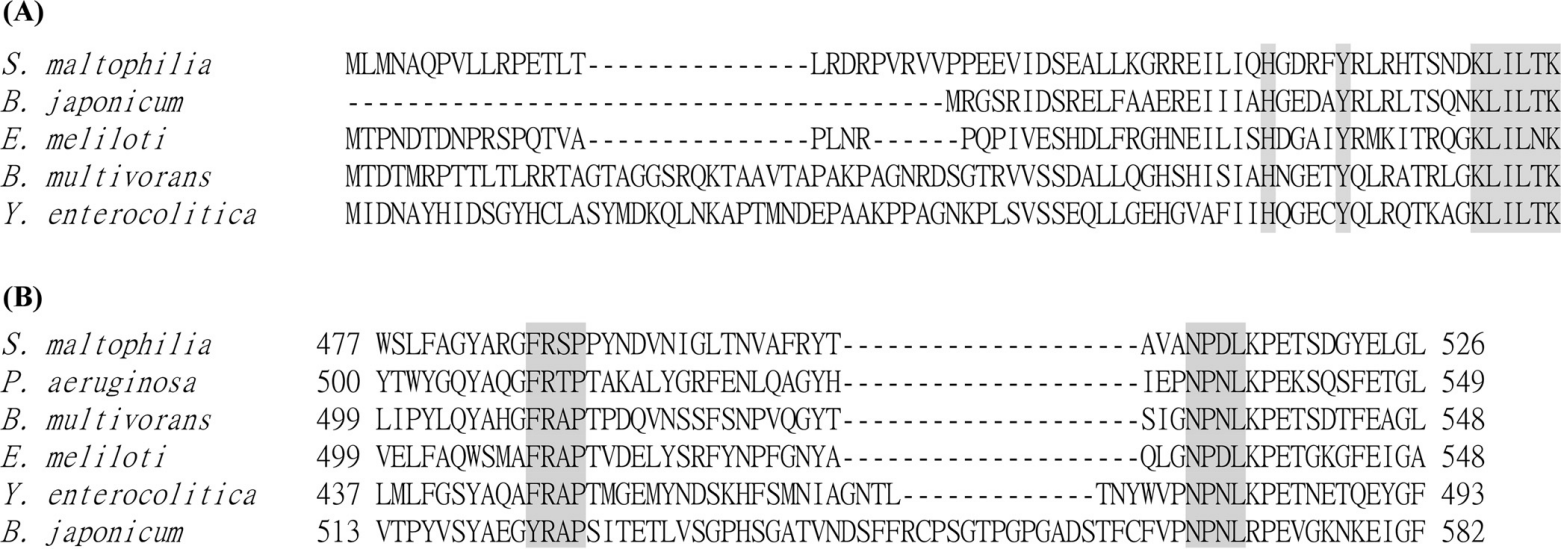
Protein alignments of HemP and HemA of S. maltophilia KJ and their homologs in other bacteria. (A) Protein alignment of HemP. The conserved KLILXK motif and histidine and tyrosine residues are marked in gray. (B) Protein alignment of HemA. The conserved FRAP and NPNL domains that coordinate hemin (Stojiljkovic et al., 1992) (17) are marked in gray.

Protein alignments of the HemP and HemA homologs were performed. Although the overall amino acid sequence similarity of the five HemP family proteins was low, a conserved KLILXK motif was found in the C terminus of S. maltophilia HemP, similar to most of those reported for HemP/HmuP proteins (18) (Fig. 4A). In addition, the C-terminal histidine and tyrosine residues, which have been shown to participate in heme binding (18), were conserved in S. maltophilia HemP (Fig. 4A). Furthermore, Fig. 4B shows the alignment of the HemA homologs, whereby the majority of the TonB-dependent outer membrane receptors share two motifs, the FRAP and NPNL domains (22), which are partially conserved in S. maltophilia HemA (Fig. 4B).

### Regulation of *hemP-hemA-smlt0796-smlt0797* operon expression.

**FIG 5:**
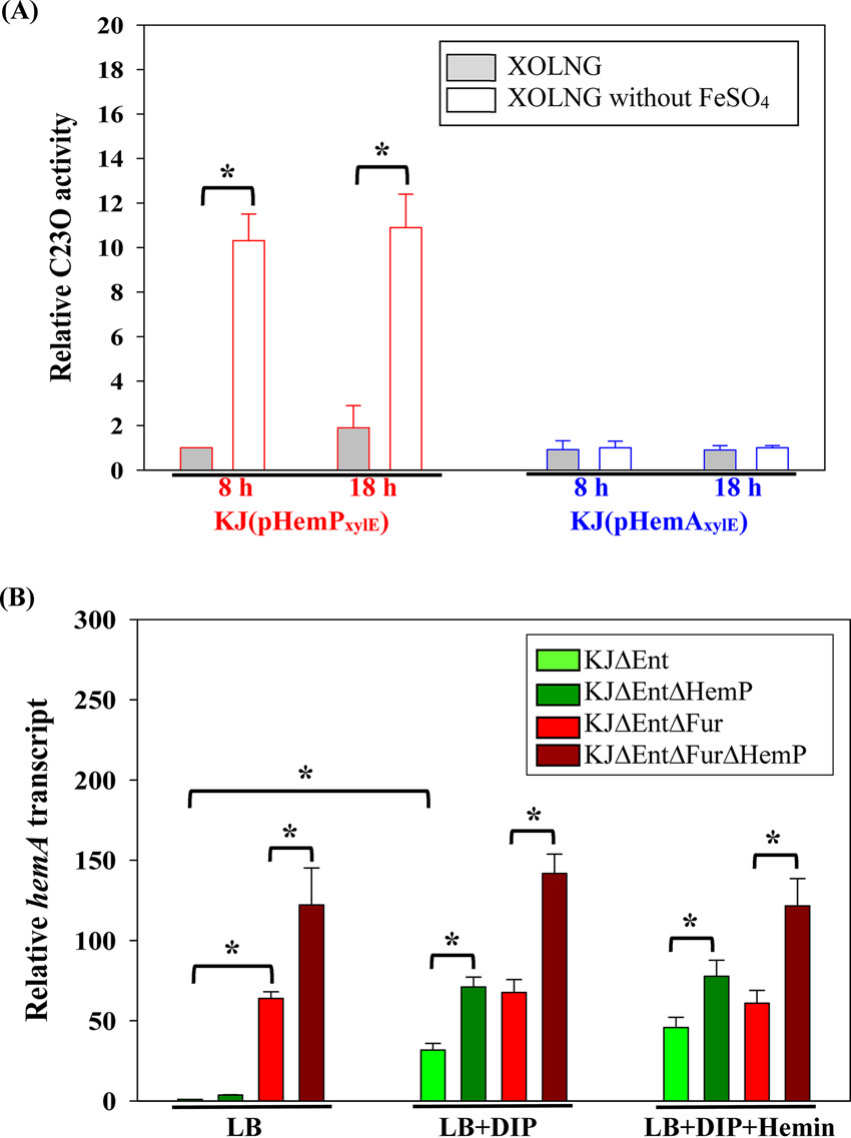
Regulation of *hemP-hemA-smlt0796-smlt0797* operon expression. Data are the means from three independent experiments. Error bars represent the standard deviations for three triplicate samples. *, *P* < 0.05 (significance calculated by Student’s test). (A) KJ(pHemP_xylE_) and KJ(pHemA_xylE_) cells were collected from a culture grown overnight and washed with FeSO_4_-free XOLNG medium to remove the residual iron in the medium. Next, the bacterial aliquot was inoculated into fresh XOLNG medium with and without FeSO_4_, respectively, at an initial OD_450_ of 0.3. Cells were grown aerobically for 8 h and 18 h before measuring the C23O activity. The relative C23O activities were calculated using the C23O activity of KJ(pHemP_xylE_) cultured in XOLNG medium for 8 h as 1. (B) Cultures of the tested S. maltophilia strains grown overnight were inoculated into the indicated fresh medium at an initial OD_450_ of 0.15. The *hemA* transcript was quantified by quantitative RT-PCR (qRT-PCR) after an 8-h incubation. The relative transcript level was calculated using the transcript level of KJ cells grown in LB broth as 1. The concentrations of DIP and hemin added were 30 μg/mL and 150 μM, respectively.

Since a 189-bp intergenic region was present between *hemP* and *hemA*, we hypothesized whether a promoter upstream of *hemA* exists. The promoter-*xylE* transcriptional fusion constructs pHemP_xylE_ and pHemA_xylE_ were prepared to test this notion (Fig. 2A and Table 1). A promoter activity assay was carried out in XOLNG minimal medium (23) with and without FeSO_4_ to assess the putative promoter activities under iron-replete and iron-depleted conditions. Meanwhile, we also investigated the impact of the growth phase on promoter activities; thus, the promoter activities were analyzed in logarithmic phase (8 h) and stationary phase (18 h). In logarithmic phase, KJ(pHemP_xylE_) displayed weak expression of the *xylE* gene in iron-replete medium and showed an ~10-fold increase in iron-depleted medium. However, KJ(pHemA_xylE_) expressed no significant catechol-2,3-dioxygenase (C23O) activity regardless of the presence of iron (Fig. 5A), indicating that the *hemP-hemA-smlt0796-smlt0797* operon is driven by the promoter upstream of *hemP*, which is active under iron-depleted conditions. By comparing the differences between the logarithmic and stationary phases, we noticed that the bacterial growth phase had an insignificant impact on *P_hemP_* promoter activity regardless of the iron levels (Fig. 5A).

**TABLE 1:** Bacterial strains and plasmids used in this study

Strain or plasmid	Property(ies) and/or genotype	Reference or source
Strains		
S. maltophilia		
KJ	Clinical S. maltophilia isolate	30
KJΔEnt	S. maltophilia KJ mutant of *entF* and *entA*; Δ*entF* Δ*entA*	21
KJΔEntΔ2937	S. maltophilia KJ mutant of *entF*, *entA*, and *smlt2937*; Δ*entF* Δ*entA* Δ*smlt2937*	This study
KJΔEntΔHemA	S. maltophilia KJ mutant of *entF*, *entA*, and *hemA*; Δ*entF* Δ*entA* Δ*hemA*	This study
KJΔEntΔHemAΔ2937	S. maltophilia KJ mutant of *entF*, *entA*, *hemA*, and *smlt2937*; Δ*entF* Δ*entA* Δ*hemA* Δ*smlt2937*	This study
KJΔEntΔHemP	S. maltophilia KJ mutant of *entF*, *entA*, and *hemP*; Δ*entF* Δ*entA* Δ*hemP*	This study
KJΔEntΔ0796	S. maltophilia KJ mutant of *entF*, *entA*, and *smlt0796*; Δ*entF* Δ*entA* Δ*smlt0796*	This study
KJΔEntΔ0797	S. maltophilia KJ mutant of *entF*, *entA*, and *smlt0797*; Δ*entF* Δ*entA* Δ*smlt0797*	This study
KJΔEntΔ0796Δ0797	S. maltophilia KJ mutant of *entF*, *entA*, *smlt0796*, and *smlt0797*; Δ*entF* Δ*entA* Δ*hemA* Δ*smlt0796* Δ*smlt0797*	This study
KJΔEntΔFur	S. maltophilia KJ mutant of *entF*, *entA*, and *fur*; Δ*entF* Δ*entA* Δ*fur*	This study
KJΔEntΔFurΔHemP	S. maltophilia KJ mutant of *entF*, *entA*, *fur*, and *hemP*; Δ*entF* Δ*entA* Δ*fur* Δ*hemP*	This study
KJΔEntΔ2355	S. maltophilia KJ mutant of *entF*, *entA*, and *smlt2355*; Δ*entF* Δ*entA* Δ*smlt2355*	This study
KJΔEntΔ2356	S. maltophilia KJ mutant of *entF*, *entA*, and *smlt2356*; Δ*entF* Δ*entA* Δ*smlt2356*	This study
KJΔEntΔ2357	S. maltophilia KJ mutant of *entF*, *entA*, and *smlt2357*; Δ*entF* Δ*entA* Δ*smlt2357*	This study
E. coli		
DH5α	F^−^ ϕ80d*lacZ*ΔM15 Δ(*lacZYA-argF*)*U169 deoR recA1 endA1 hsdR17*(r_K_^−^ m_K_^+^) *phoA supE44*λ *thi-1 gyrA96 relA1*	Invitrogen
S17-1	λ*pir*^+^ mating strain	35

Plasmids		
pEX18Tc	*sacB oriT*; Tc^r^	36
pRK415	Mobilizable broad-host-range plasmid cloning vector, RK2 origin; Tc^r^	37
pRKXylE	pRK415-derived vector for construction of the promoter-*xylE* transcriptional fusion; the orientation of the *xylE* gene in this plasmid is opposite that of *P_lacZ_* of pRK415; Tc^r^	34
pHemA	pRK415 with an intact *hemA* gene; Tc^r^	This study
pHemP_xylE_	pRK415 with a 559-bp DNA fragment upstream from the *hemP* start codon and a *P_hemP_*::*xylE* transcriptional fusion	This study
pHemA_xylE_	pRK415 with a 220-bp DNA fragment upstream from the *hemA* start codon and a *P_hemA_*::*xylE* transcriptional fusion	This study
pΔ2937	pEX18Tc with an internally deleted *smlt2937* gene; Tc^r^	This study
pΔHemA	pEX18Tc with an internally deleted *hemA* gene; Tc^r^	This study
pΔHemP	pEX18Tc with an internally deleted *hemP* gene; Tc^r^	This study
pΔ0796	pEX18Tc with an internally deleted *smlt0796* gene; Tc^r^	This study
pΔ0797	pEX18Tc with an internally deleted *smlt0797* gene; Tc^r^	This study
pΔ2355	pEX18Tc with an internally deleted *Smlt2355* gene; Tc^r^	This study
pΔ2356	pEX18Tc with an internally deleted *Smlt2356* gene; Tc^r^	This study
pΔ2357	pEX18Tc with an internally deleted *Smlt2937* gene; Tc^r^	This study

Next, we investigated the role of Fur in regulating the expression of the *hemP-hemA-smlt0796-smlt0797* operon using the XOLNG minimal medium system; nevertheless, the *fur* deletion mutant (KJΔEntΔFur) grew poorly in XOLNG medium. We alternatively used LB medium with and without DIP to represent the iron-depleted and iron-replete conditions, respectively. Under iron-replete conditions, the level of the *hemA* transcript of KJΔFur had a 63.9-fold ± 4.1-fold increase compared to that of KJ cells (Fig. 5B), indicating that the *hemP-hemA-smlt0796-smlt0797* operon is negatively regulated by Fur. The putative “Fur box” of S. maltophilia (24) was found upstream of the *hemP-hemA-smlt0796-smlt0797* operon (Fig. 2C). In response to DIP treatment, the *hemA* transcript level increased in KJ cells but not in KJΔFur cells (Fig. 5B).

To assess the role of HemP in *hemP-hemA-smlt0796-smlt0797* operon expression, we performed a pairwise comparison of *hemA* transcripts of KJ and KJΔHemP as well as KJΔFur and KJΔFurΔHemP. The assayed conditions included iron-replete medium (LB medium), iron-depleted medium (LB medium plus DIP), and iron-depleted medium supplemented with hemin (LB medium plus DIP and hemin). The inactivation of *hemP* resulted in an ~2-fold increase in the *hemA* transcript level in a *fur* mutant background and/or under DIP-treated conditions (Fig. 5B).

To further assess the roles of Fur and HemP in hemin acquisition under iron-depleted conditions, the viabilities of KJΔEnt, KJΔEntΔHemP, KJΔEntΔFur, and KJΔEntΔFurΔHemP in DIP- and hemin-supplemented media were evaluated. The viability of KJΔEntΔHemP was slightly, but not significantly, better than that of KJΔEnt in DIP- and hemin-supplemented media (Fig. 1C). However, compared to KJΔEnt, KJΔEntΔFur displayed compromised viability in DIP- and hemin-supplemented media, and this compromise was attenuated when the Δ*hemP* mutation was introduced into the chromosome of KJΔEntΔFur (Fig. 1C). Thus, HemP appears to exert a negative impact on hemin utilization in a *fur* mutant but not in the parental strain background.

### Significance of the *hemP-hemA-smlt0796-smlt0797* operon in hemin acquisition when siderophore-mediated iron uptake is active.

To understand the significance of *hemP-hemA-smlt0796-smlt0797* when siderophore-mediated iron uptake is active, we constructed a set of mutants in the wild-type KJ background, yielding KJΔHemP, KJΔHemA, KJΔ0796, and KJΔ0797, and a hemin utilization assay was carried out. KJΔHemA, but not KJΔHemP, KJΔ0796, and KJΔ0797, displayed compromised growth in iron-depleted medium supplemented with hemin (see Fig. S1 in the supplemental material), similar to the phenotype observed in strains of a stenobactin-null background (Fig. 1B). This observation implies that the affinity of stenobactin for ferric iron is not high enough to sequester ferric iron from hemin, signifying the importance of HemA in iron acquisition when hemin is the sole iron source available.

### The putative HmuV, HmuU, and HmuT homologs (Smlt2357, Smlt2356, and Smlt2355, respectively) are not involved in hemin acquisition.

Once hemin is taken up by HemA and transported into the periplasmic space, a hemin cytoplasmic membrane permease is required for the transport of hemin into the cytosol. Kalidasan et al. conducted a genome-wide analysis of putative iron acquisition systems of S. maltophilia (14). Based on their analysis, Smlt2357, Smlt2356, and Smlt2355 are homologs of *hmuV*, *hmuU*, and *hmuT*, respectively, which are known inner membrane transporters of hemin in other bacteria (14). Thus, the involvement of Smlt2357, Smlt2356, and Smlt2355 in hemin acquisition was investigated. The Δ*smlt2357*, Δ*smlt2356*, and Δ*smlt2355* alleles were introduced into the chromosome of KJΔEnt to generate the mutants KJΔEntΔ2357, KJΔEntΔ2356, and KJΔEntΔ2355. All three mutants displayed viability comparable to that of the parental strain in iron-depleted medium supplemented with hemin (Fig. 1D), suggesting that Smlt2357, Smlt2356, and Smlt2355 are not individually critical for hemin acquisition in S. maltophilia.

## DISCUSSION

In most of the known hemin acquisition systems, the genes associated with the transport of hemin across both the outer and inner membranes are generally clustered, such as those of Y. enterocolitica, P. aeruginosa, B. japonicum, and *B. multivorans* (17–20) (Fig. 3A). However, in S. maltophilia, *hemA*, which encodes the TonB-dependent outer membrane receptor, is clustered with *hemP*, but these genes do not cluster with the genes associated with the transport of hemin across the inner membrane.

Unlike most gammaproteobacteria, but like alpha- and betaproteobacteria, S. maltophilia, a member of the *Gammaproteobacteria*, contains the *hemP* gene. HemP/HmuP is a well-known transcriptional activator essential for the expression of hemin uptake TonB-dependent outer membrane receptors in alpha- and betaproteobacteria (17–20). However, in this study, our results suggested that HemP of S. maltophilia acts as a transcriptional repressor, having a negative effect on the *hemA* transcript level. To our knowledge, the negative regulatory role of S. maltophilia HemP represents a novel property that has not been observed in other HemP superfamily proteins.

Based on the established hemin acquisition systems in other Gram-negative bacteria, hemin is bound to the TonB-dependent OMP and then taken to the periplasmic space. Iron complexes are transported from the periplasm to the cytoplasm through ABC transporters. The ABC transport system generally consists of an inner membrane hemin permease, a cytoplasmic hemin-binding protein, a cytoplasmic hemin import ATP-binding protein, and a cytoplasmic heme degradation protein (Fig. 3B) (25). Interestingly, unlike FecB and FepB in the ferric citrate and ferrienterobactin acquisition systems of Escherichia coli (26, 27), no periplasmic hemin-binding proteins were reported in these known hemin acquisition systems (Fig. 3B). These hemin acquisition-associated genes are frequently located in the same operon as or near outer membrane receptor genes. We therefore predicted that Smlt0796 and Smlt0797 are members of the ABC transport system for hemin acquisition in S. maltophilia. However, our results did not support this but rather supported that Smlt0796 and Smlt0797 are not individually crucial for hemin acquisition in S. maltophilia (Fig. 1B). Similar observations were also reported for the *hemPRST* cluster of Y. enterocolitica (Fig. 3A), in which *hemP*, *hemR*, and *hemS* participate in hemin acquisition but *hemT* is not necessary for hemin uptake (17). To obtain insight into their putative functions, we assessed the homology of Smlt0796 and Smlt0797 with known proteins. Nevertheless, no positive results were obtained. Thus, the exact functions of Smlt0796 and Smlt0797 are still unclear at present.

Interestingly, under iron-depleted and hemin-supplemented conditions, HemP functioned as a transcriptional factor negatively regulating the expression of the *hemA* transcript in either a parental strain (KJΔEnt) or a *fur* mutant (KJΔEntΔFur) (Fig. 5B). However, with respect to hemin utilization under iron-depleted conditions, the negative impact of HemP on hemin utilization was observed in a *fur* mutant (KJΔEntΔFur) but not in a parental strain (KJΔEnt) (Fig. 1C). By further inspecting the results shown in Fig. 1C and Fig. 5B, we noticed that the *hemA* transcript level of KJΔEnt in DIP- and hemin-supplemented LB medium had a 45.8-fold ± 6.3-fold increase compared to that in LB medium (Fig. 5B), and KJΔEnt was able to utilize hemin as the sole iron source to support growth under iron-depleted conditions (Fig. 1C). Thus, the *hemA* upregulation level in KJΔEnt should be enough to support hemin utilization. However, even though the *hemA* transcript level of KJΔEntΔFur was higher than that of KJΔEnt (Fig. 5B), the viability of KJΔEntΔFur was worse than that of KJΔEnt in DIP- and hemin-supplemented medium (Fig. 1C). HemA is prerequisite for hemin utilization; nevertheless, functional periplasmic and cytoplasmic hemin transport system components are required for hemin utilization. Based on this rationale, the underlying mechanism responsible for the compromised hemin utilization of KJΔEntΔFur may be the defect in periplasmic or cytoplasmic hemin transport components rather than HemA. Furthermore, our results also supported that the Δ*fur-*mediated reduction in hemin utilization under iron-depleted conditions can be HemP dependent since KJΔEntΔFurΔHemP displayed viability comparable to that of KJΔEnt in DIP- and hemin-supplemented media (Fig. 1C).

HemP (or HmuP) functions as a transcriptional activator and is essential for the expression of hemin uptake outer membrane receptors, which has been reported in Y. enterocolitica, B. japonicum, *E. meliloti*, and *B. multivorans* (17–20). Distinct from these systems, S. maltophilia HemP negatively regulated the expression of *hemA* in a *fur* mutant and/or under iron-depleted conditions (Fig. 5B). This discovery led us to speculate whether the HemP-binding region overlaps or is near the Fur box; thus, Fur-Fur box binding sequesters the opportunity for HemP binding. It has been proposed that HemP/HmuP specifically binds to a conserved DNA motif present upstream of the genes encoding the heme outer membrane receptor. The conserved DNA motif is named as a HmuP-responsive element in *E. meliloti*, an alphaproteobacterium (28), and is assigned as a HemP-binding region in *B. multivorans*, a betaproteobacterium (20). The HmuP-responsive element is a direct repeat motif that is widely distributed among alpha- and betaproteobacteria but is absent in gammaproteobacteria (28). In *B. multivorans*, the HemP-binding region of an inverted repeat frame was revealed by Sato et al. (20). Thus, we inspected the promoter-containing region of the *hemP-hemA-smlt0796-smlt0797* operon and found an inverted repeat sequence (underlined), TGATTGTCAACTGAGAATCA, although it was not similar to the HemP-binding region of *B. multivorans*, GGCTGCGCGCCCGGGCGCAGCC (20). This is highly likely to be the HemP-binding region for HemP in S. maltophilia (Fig. 2C) based on the rationale that the binding motif of HemP/HmuP has a direct repeat or an inverted repeat feature. The putative HemP-binding region partially overlaps the Fur box, providing an explanation for the repressor role of HemP in *hemA* expression in a *fur* mutant and/or under iron-depleted conditions. Although hemin is an important iron source for invading bacteria, excess hemin is also very toxic to bacteria, most likely due to the generation of hydroxyl radicals by the heme-mediated Fenton reaction (29). The two-stage negative regulatory circuit for hemin acquisition by Fur and HemP emphasizes the importance of the optimal expression of the hemin acquisition system.

## MATERIALS AND METHODS

### Bacterial strains, media, plasmids, and primers.

The bacterial strains and plasmids used in this study are listed in Table 1. S. maltophilia KJ has been used as a wild-type strain in our previous series of studies since 2008 (30), and an array of isogenic deletion mutants of S. maltophilia KJ is available in our stock. The complete genome sequence of S. maltophilia KJ is not available at present. Nevertheless, based on our previous experience, the genome of the KJ strain is highly similar to that of the K279a strain (31) regarding gene organizations and DNA sequences. Thus, we chose S. maltophilia K279a as a reference strain for bioinformatics analysis and PCR primer design in this study.

Luria-Bertani (LB) medium and XOLNG medium were used as nutrient and minimal media, respectively. XOLNG medium was prepared as described previously (23). XOLNG medium without FeSO_4_·7H_2_O was used as iron-depleted minimal medium. Cells were grown at 37°C with shaking in broth. Hemin (150 μM), DIP (50 μg/mL), and tetracycline (10 μg/mL) were added when required.

### Mutant construction.

S. maltophilia chromosomal in-frame deletion mutants were generated by allelic replacement using the suicide vector pEX18Tc, as previously described (32). Briefly, two DNA fragments containing ~500 bp of the N terminus and C terminus of the intended deletion region were amplified from S. maltophilia KJ by PCR using the designed primer pairs. The primer pairs used are listed in Table S1 in the supplemental material. The PCR amplicons were subsequently cloned into pEX18Tc, generating recombinant plasmids pΔ2397, pΔHemA, pΔHemP, pΔ0796, pΔ0797, pΔ2355, pΔ2356, and pΔ2357 (Table 1). To delete each target gene, the pEX18Tc-derived recombinant plasmids were transferred to S. maltophilia KJ by conjugation. Transconjugant selection, double-crossover mutant selection, and confirmation were carried out as described previously (32). The correctness of mutants was confirmed by PCR (Fig. S2) and DNA sequencing. Double and triple mutants were constructed from the single mutant sequentially using the same procedure.

### Hemin utilization assay.

A hemin utilization assay was designed to investigate whether the assayed strain under iron-depleted conditions can utilize exogenous hemin as the iron source for growth. During LB agar preparation, 50 μg/mL DIP was added to create iron-depleted conditions (21). If needed, 150 μM hemin (Sigma-Aldrich) was supplemented as an iron source. Logarithmic-phase bacterial cells tested at 2 × 10^5^ CFU/μL were 10-fold serially diluted. Five microliters of the bacterial suspension was spotted onto LB agar containing 50 μg/mL DIP and 150 μM hemin. After a 24-h incubation at 37°C, bacterial viability was imaged.

### Construction of complementation plasmid pHemA.

The *hemA* complementation plasmid pHemA was constructed by amplifying *hemA* from S. maltophilia KJ with primers HemA-F and HemA-R and cloning into pRK415 under the control of the vector *lacZ* promoter. The *lacZ* promoter of pRK415 was constitutively active in S. maltophilia.

### Reverse transcriptase PCR.

DNA-free RNA preparation, reverse transcriptase PCRs (RT-PCRs), and data analysis were performed as described previously (33). S. maltophilia KJΔFur cultured overnight was inoculated into fresh LB medium at an initial optical density at 450 nm (OD_450_) of 0.15 and grown for 5 h. DNA-free RNA isolated from KJΔFur cells was reverse transcribed using the primer 0797-C (Table S1). The 0797-C-derived cDNA was used as a template for PCR using primers sets HemPQ95-F/R, HemAQ93-F/R, 0796Q120-F/R, and 0797Q108-F/R (Table S1). The 0797Q108-F/R primer set targeted the region downstream of the 0797-C primer and within the C terminus of the *smlt0797* gene, which was used as a negative control to rule out DNA contamination.

### Construction of promoter-*xylE* transcriptional fusions.

The DNA segments containing the upstream 559-bp region of *hemP* and the upstream 220-bp region of *hemA* were amplified by PCR using the primer sets HemAN-F/R and HemPN-F/HemPN-R and cloned into plasmid pRKXylE (34) with compatible restriction enzyme sites, yielding pHemP_xylE_ and pHemA_xylE_, respectively. The correctness of the orientation of the cloned fragments was confirmed by DNA sequencing.

### C23O activity determination.

The activities of catechol-2,3-dioxygenase encoded by the *xylE* gene were measured as described previously (34). Each assay was run in duplicate at least three times, and the data given are averages.

### Bioinformatics assay.

A protein homolog search was carried out using the BLASTp tool from the NCBI (https://blast.ncbi.nlm.nih.gov/Blast.cgi) against the S. maltophilia K279a genome (GenBank accession no. AM743169.1).

### Statistical analysis.

Student’s *t* test was used for comparison of means between the groups. The Bonferroni correction method was applied to adjust the *P* values.
