# Supplementary material for: Involvement of the hemP-hemA-smlt0796-smlt0797 Operon in Hemin Acquisition by Stenotrophomonas maltophilia
Source: Microbiol Spectr. 2022 Jun 6;10(3):e00321-22. doi: 10.1128/spectrum.00321-22 (PMC9241770; doi:10.1128/spectrum.00321-22)
Supplement: Supplemental file 1 — Supplemental material. Download spectrum.00321-22-s0001.pdf, PDF file, 0.3 MB [file spectrum.00321-22-s0001.pdf]

Table S1 PCR primers used in this study[a]

Primer	Sequence (5'→3')	Purpose
2937N-F<br>2937N-R	GTGAGCTCTACCGAGCATCG<br>CTGGTACCAGTGGAACGGTG	pΔ2937 construction
2937C-F<br>2937C-R	GGGGTACCATCGAAAACCTG<br>CATTCTAGAAGTTCCTGCAGG	pΔ2937 construction
HemAN-F<br>HemAN-R	GAAGAGCTCATCGACAGCGAA<br>CGGGTACCGAAACCATCCAG	pΔHemA construction
HemPN-F<br>HemPN-R	CTCAAGCTTAAGTACGACCAGGGCATCC<br>GTTTCTAGACGCAGCAGTACAGGTTGAGCA	pΔHemP & pΔHemA construction
0796N-F<br>0796N-R	GCGGTACCTCGAATGGTCGT<br>CGTCTAGAGGGTCGACAGAC	pΔHemP & pΔ0796 construction
0797N-F<br>0797N-R	CACTCTAGACGCCAGTCTGTCGACCCTGTCCT<br>CGTGAGCTCGACCACGATGACCACTTCACTG	pΔ0796 & pΔ0797 construction
0797C-F<br>0797C-R	CAAGAGCTCGGAAAAGGCCCAGTAGATCC<br>GCAGAATTCGTGAAGCTGGTGATGGTGTTC	pΔ0797 construction
2355N-F<br>2355N-R	GTGGTCGCCAATCTTGATG<br>CTGGTATCGGCCACTTCAAT	pΔ2355 construction
2356N-F<br>2356N-R	CAGGTACCACGACTGATCCA<br>CATCTAGACCAGCAACATCGT	pΔ2355 & pΔ2356 construction
2357N-F<br>2357N-R	CGTCTAGATTCGTTGGCCTGGT<br>AGAAGCTTGCCCAACAGGCT	pΔ2356 & pΔ2357 construction
2357C-F<br>2357C-R	GTGCTGCACGACCTCAATC<br>CACCGTCGCTACTGGAATG	pΔ2357 construction
HemA-F<br>HemA-R	CATCTAGAAGCTGATCCTGA<br>GCTCATGAGCTCACCAGGAA	pHemA construction
0797-C	CTCGACCACGATGACCACTTCACTG	RT-PCR
HemPQ95-F<br>HemPQ95-R	GAATGCTCAACCTGTACTGCTG<br>CTTCGCTGTCGATGACCTCT	RT-PCR
HemAQ93-F<br>HemAQ93-R	CCTGCTCAGCAAACTGGTCT<br>AGCACATTGGTATCGGTGGT	RT-PCR
0796Q120-F<br>0796Q120-R	ACCGTGTTGTGCCTGTACCA<br>AAGGAAGCACAGGCTTTC	RT-PCR
0797Q108-F<br>0797Q108-R	GTGCACCTGAAGACCAGGAT<br>GTCGTCGATCTGCTTGTCCT	RT-PCR
2937m-F<br>2937m-R	GACCAGTTGGCCACTCAGTT<br>AGTCCAGCAGTTCCTGCAGG	KJΔ2937 mutant check

HemAm-F<br>HemAm-R	GAAGAGCTCATCGACAGCGAA<br>CGTCTAGAGGGTCGACAGAC	KJΔHemA mutant check
HemPm-F<br>HemPm-R	AAGTACGACCAGGGCATCC<br>CGGATGCGGAAACCATCCAG	KJΔHemP mutant check
0796m-F<br>0796m-R	GCTACATCGAATGGTCGTC<br>CGGTGTACTCACCCTGCAT	KJΔ0796 mutant check
0797m-F<br>0797m-R	CGCCAGTCTGTCGACCCTGTCCT<br>GTGAAGCTGGTGATGGTGTTC	KJΔ0797 mutant check
2355m-F<br>2355m-R	ATCACCCCGAAGCAGTAAGTC<br>GGAATACGCAGCTGCCAGAC	KJΔ2355 mutant check
2356m-F<br>2356m-R	CTTGCAGCTGAAGTACACC<br>CCACCTCATGCAGCTTCAAC	KJΔ2356 mutant check
2357m-F<br>2357m-R	CGTTCTTCCTCTGGCTGGT<br>GACCAGCGAGGCGTTCTC	KJΔ2357 mutant check

[a]The restriction enzyme sites were underlined

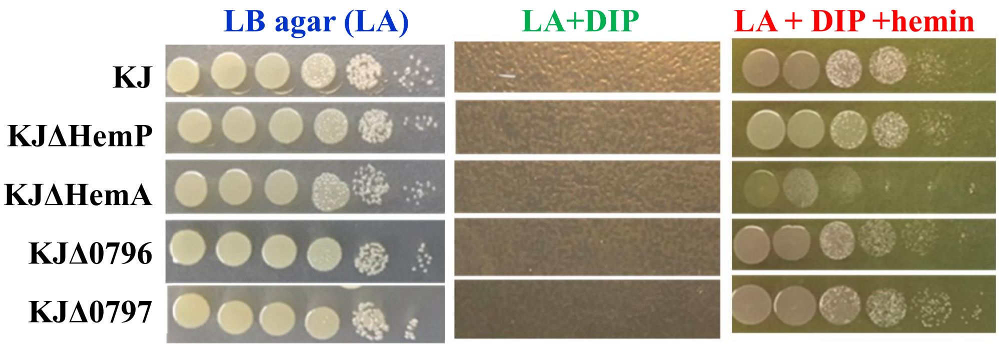

Fig. S1. The impact of hemin uptake system on a siderophore-proficient strain. The logarithmic-phase bacterial cells tested of 2 × 10^5 CFU/µl were 10-fold serially diluted. Five microliters of bacterial suspension were spotted onto the LB agar plates as indicated. The growth of bacterial cells was recorded after 24-h incubation at 37°C. The concentrations of DIP and hemin added are 50 µg/ml and 150 µM, respectively.

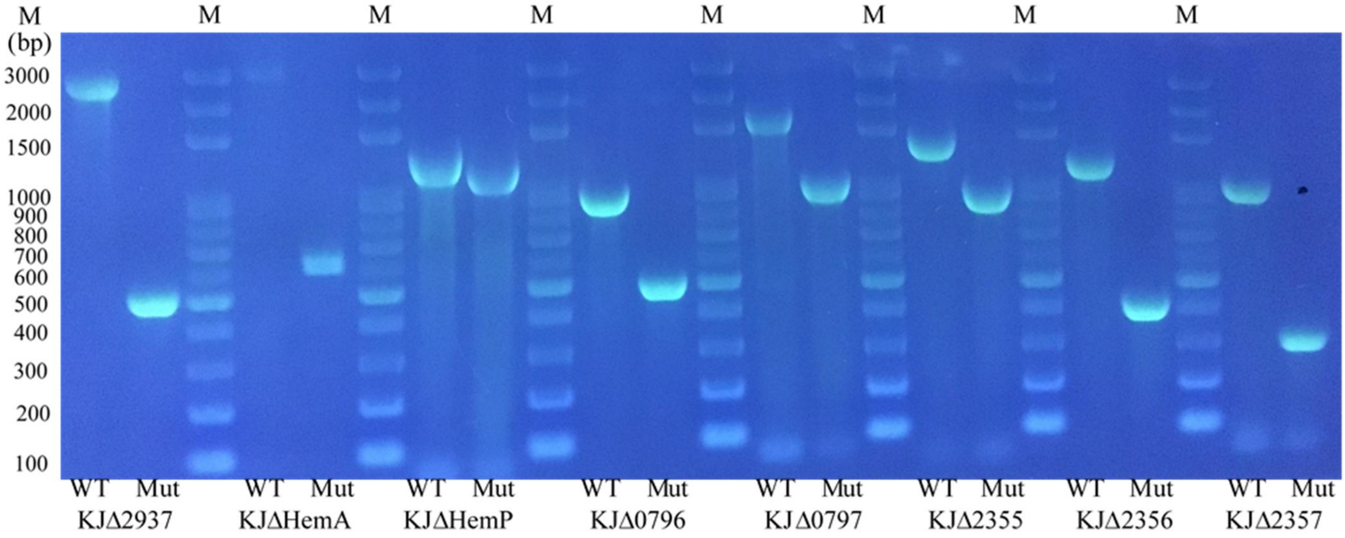

Fig. S2. Confirmation of single in-frame deletion mutants by PCR. The deletion regions of in-frame deletion mutants of *S. maltophilia* KJ were checked by colony PCR using the primer pairs, which targeted onto the upstream and downstream the deleted region, respectively. In the meanwhile, the wild-type KJ was also included for comparison. The primers used were listed in the Table S1. The PCR amplicons were separated by agarose gel electrophoresis and visible by ethidium bromide staining. Abbreviations: WT, wild-type; Mut, deletion mutant; M, DNA ladder.
